# Development and validation of a prognostic nomogram for predicting post-operative pulmonary infection in gastric cancer patients following radical gastrectomy

**DOI:** 10.1038/s41598-019-51227-4

**Published:** 2019-10-10

**Authors:** Haifan Xiao, Huijun Zhou, Ke Liu, Xianzhen Liao, Shipeng Yan, Bin Yin, Yongzhong Ouyang, Hua Xiao

**Affiliations:** 1 Department of Cancer Prevention and Control, Hunan Cancer Hospital and the Affiliated Cancer Hospital of Xiangya School of Medicine, Central South University, Changsha, People’s Republic of China; 2 Department of Gastroenterology and Urology, Hunan Cancer Hospital and the Affiliated Cancer Hospital of Xiangya School of Medicine, Central South University, Changsha, People’s Republic of China; 3 Department of Lamphoma and Abdominal Radiotherapy, Hunan Cancer Hospital and the Affiliated Cancer Hospital of Xiangya School of Medicine, Central South University, Changsha, People’s Republic of China; 4 Department of Gastroduodenal and Pancreatic Surgery, Hunan Cancer Hospital and the Affiliated Cancer Hospital of Xiangya School of Medicine, Central South University, Changsha, People’s Republic of China

**Keywords:** Cancer models, Gastric cancer, Cancer prevention

## Abstract

The aim of this retrospective study was to develop and validate a nomogram for predicting the risk of post-operative pulmonary infection (POI) in gastric cancer (GC) patients following radical gastrectomy. 2469 GC patients who underwent radical gastrectomy were enrolled, and randomly divided into the development and validation groups. The nomogram was constructed based on prognostic factors using logistic regression analysis, and was internally and crossly validated by bootstrap resampling and the validation dataset, respectively. Concordance index (C-index) value and calibration curve were used for estimating the predictive accuracy and discriminatory capability. Sixty-five (2.63%) patients developed POI within 30 days following surgery, with higher rates of requiring intensive care and longer post-operative hospital stays. The nomogram showed that open operation, chronic obstructive pulmonary disease (COPD), intra-operative blood transfusion, tumor located at upper and/or middle third and longer operation time (≥4 h) in a descending order were significant contributors to POI risk. The C-index value for the model was 0.756 (95% CI: 0.675−0.837), and calibration curves showed good agreement between nomogram predictions and actual observations. In conclusion, a nomogram based on these factors could accurately and simply provide a picture tool to predict the incidence of POI in GC patients undergoing radical gastrectomy.

## Introduction

Gastric cancer (GC) is the fifth most common cancer and the third leading cause of cancer-related death according to the Global Cancer Statistics 2018^1^. For GC patients, resection offers the only potential curative treatment to date. Although mortality and morbidity following radical gastrectomy for GC have reduced significantly with improvements of surgical techniques and peri-operative managements, post-operative pulmonary complications (PPCs), including post-operative pulmonary infection (POI), remain a clinically important event, especially among older or immuno-compromised patients^2^. With an incidence rate ranging from 1.1–12.3%^3–7^, PPCs have been identified to be associated with the largest attributable healthcare costs and hospital stays; even with increased peri-operative death^8,9^. Moreover, there is a growing body of evidence that post-operative infectious complications, including POI, is an adverse predictor for prognosis in various of cancer patients^10–15^. Thus, to reduce POI, it is essential to clarify the risk factors and to identify those patients at greatest risk of POI, and allow early intervention to reduce its incidence or improve post-operative clinical outcomes. Although several studies have investigated the risk factors for POI following gastrectomy for GC, their conclusions were usually based on a limited number of patients and have never been externally validated^15,16^.

Using a graphical calculator, nomograms have been used to build predictive models that serve as popular mathematical tools. There is a growing body of evidence that a nomogram can predict the metastatic probability, recurrence probability, overall survival, and disease risk for most types of cancer^17^. In addition, these nomograms have served as intuitive and simple tools for clinicians to reduce disease risk or improve prognosis by tailoring clinical treatments to the needs of individual patients. But to our knowledge, there has never been a study intended to develop a nomogram to predict the risk of POI following radical gastrectomy. Therefore, in this retrospective study, for the first time, we developed and cross-validated a novel nomogram to predict POI in GC patients following curative resection, using the database from a high volume tertiary hospital in China.

## Patients and Methods

### Patient population

**Figure 1:**
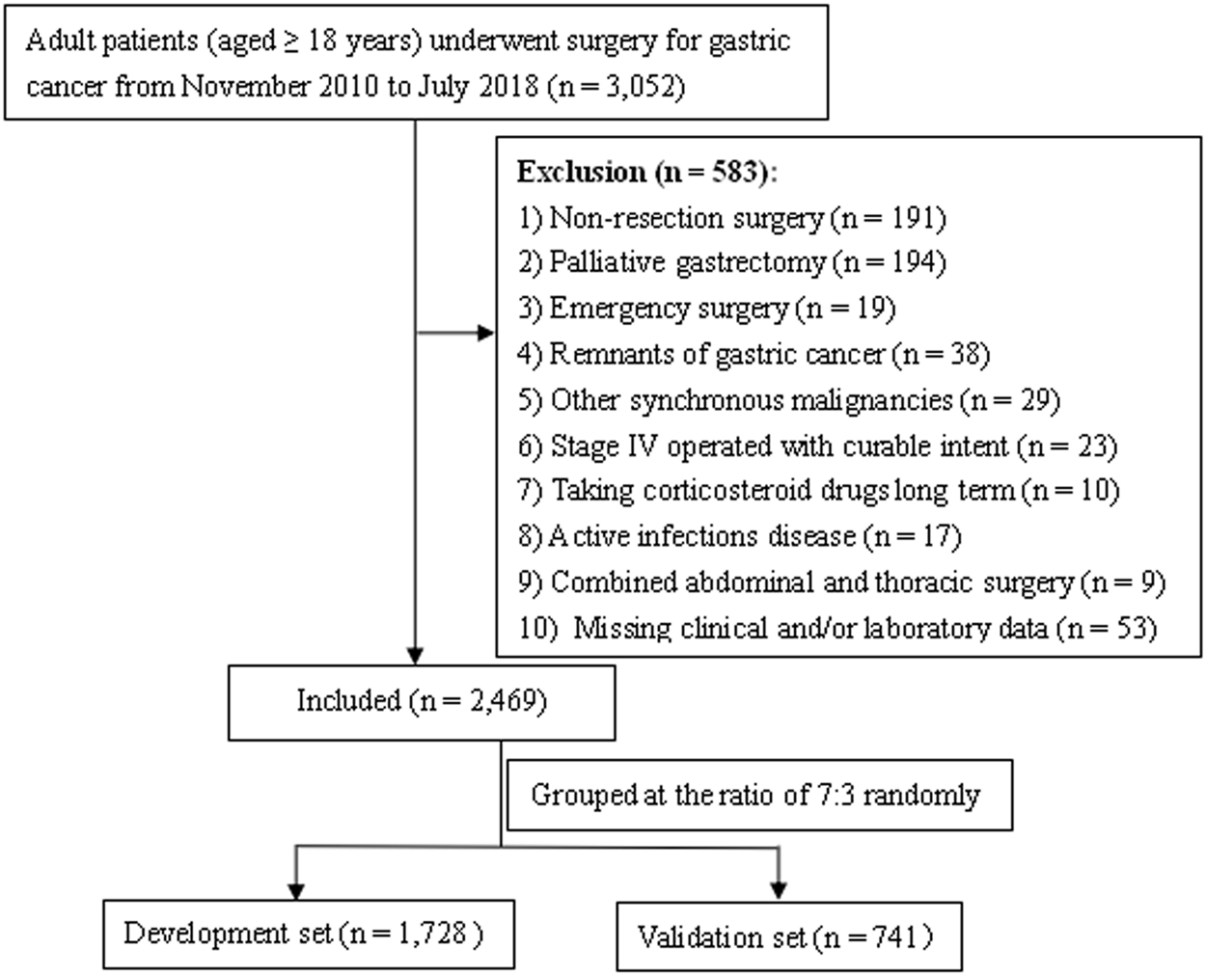
Flow-chart.

We reviewed the prospectively registered data of adult patients diagnosed with GC and underwent curative gastric resection in Hunan Cancer Hospital between November 2010 and July 2018. The inclusion and exclusion criteria are described in Figure 1. In total, 2469 consecutive patients were included in our study. Ethical approval was obtained by the Institutional Review Board of our hospital (N0.17 scientific research quick review in 2018). Every patient provided written informed consent for surgery and the use of their clinical data. To test the generalizability of the model, patients were randomly assigned to the development group (n = 1728) and the validation group (n = 741) at a ratio of 7:3.

### Peri-operative management and surgical procedures

Prior to surgery, all patients were subjected to laboratory examination, abdominal computed tomography (CT), as well as X-ray and/or CT of the chest. The curative gastrectomy and lymphadenectomy was performed by qualified physicians according to the Japanese gastric cancer treatment guidelines^18^. As described in our previous study^19^, open procedures were usually performed for D2 or D2 + lymphadenectomy in patients with advanced GC, and laparoscopy or laparoscopy-assisted gastrectomy with D1 + α or β lymphadenectomy were performed for patients with early GC. To achieve R0 resection margins, combined multiorgan resection was performed in patients with a locally advanced tumor with potential invasion of nearby organs and in those with simultaneously occurring benign disease in other organs. All patients received a second- or third-generation cephalosporin for 3 to 5 days following surgery as prophylactic antibiotic treatment. Platinum and fluorouracil based regimens, such as S-1 plus oxaliplatin, were used for neoadjuvant chemotherapy, in some patients with stage cT3-4N + GC.

### Data collection

The clinicopathological factors including baseline demographics, pre-operative laboratory measurements, intra-operative variables and pathological tumor characteristics (based on American Joint Committee on Cancer TNM staging system 7th edition) were evaluated. The primary outcome assessed was the development of POI within 30 days following surgery. The post-operative morbidity during the 30-day follow-up period was checked by the same physician during hospital stay. We sent an e-mail or letter or gave a call to patients who did not report for follow-up examinations. Given their little clinical significance of stage I complications, only Clavien-Dindo grade II or higher were regarded as having complications in the present study^19^.

Patients with lung infiltrate visible in chest X-ray and/or CT scan combined with respiratory symptoms, fever, leukopenia, or leukocytosis were diagnosed with POI, either following other complications (such as anastomotic leakage) or not, according to the criteria proposed by the United States Centers for Disease Control and Prevention^20^. The diagnosed with POI was checked by the researchers of the present study again by carefully reviewing the medical records of each patient.

### Statistical analysis

We use univariate logistic analysis to analyze risk factors. All variables were entered into the multivariate logistic regression model through a backward step-down process performed using the Akaike information criterion stopping rule. The nomogram in our study was designed to give the risk probability of POI. Parameter estimates obtained from the above the multivariate logistic regression were used to construct nomogram scale, and only the variable with *P* value less than 0.05 were incorporated into the nomogram. Every variable in regression model can get a score on the top point line (0–100 points by default) in the nomogram plot thought drawing corresponding vertical line, then a total score can be get by adding every variable’s score. Last, we can obtain the predicted risk value through drawing a vertical line from the total score to the bottom risk line.

Model validation included bootstrap self-sample validation and cross validation. Bootstrap self-sample validation was performed by using 1000 bootstrap resamples of the development dataset. Source data was divided into two sets, the bigger set was used for developing the model while the smaller set was applied to the validation the constructed model, which called cross validation. The prognostic performance of the model was evaluated based the concordance index (C-index) value on a scale from 0 to 1 and 95% confidence interval (CI), as well as the area under the curve (AUC) for the receiver operator characteristic (ROC) plot. Generally, a C-index value > 0.70 indicated that the model was good for discrimination. The nomogram was calibrated by comparing the predicted rates of POI with the observed rates. All data management and statistical analyses were performed using the R software, version 3.5.1 (R Foundation for Statistical Computing, Vienna, Austria). All statistical tests were two-sided, and a *P*-value < 0.05 was considered statistically significant.

### Ethical approval

All procedures performed in studies involving human participants were in accordance with the ethical standards of the institutional and/or national research committee and with the 1964 Helsinki declaration and its later amendments or comparable ethical standards.

## Results

### Clinicopathological characteristics and incidence of POI

The clinicopathological characteristics of the 2469 patients are shown in Table 1. The cohort included 1624 men (65.78%) and 845 women (34.22%), with a mean age as 55.44 years (range, 19–86). Of whom, 1888 patients (76.47%) were performed subtotal gastrectomy, and the remaining 581 cases underwent total gastrectomy. The mean operation time was 199.28 min (range, 70–584), and the mean estimated intra-operative blood loss was 203.41 mL (range, 30–2300). According to the 7th edition of the TNM staging system for GC, there were 649 (26.29%) stage I, 514 (20.82%) stage II, and 1306 (52.90%) stage III patients.

**Table 1:** Clinicopathologic characteristics of patients and univariate logistic analysis in patients.

Clinicopathological parameters		Development set (n = 1728)					Validation set (n = 741)				
		N	Pulmonary infection		OR (95% CI)^d^	*P*	N	Pulmonary infection		OR (95% CI)^d^	*P*
			Negative	Positive				Negative	Positive		
ALL		1728	1688	40			741	716	25		
Gender	Male	1154	1124	30	1.51 (0.78–2.23)	0.267	470	455	15	0.86 (0.05–1.68)	0.718
	Female	574	564	10	Ref		271	261	10	Ref	
Age ($\bar{{\rm{\chi }}}$ ± S)			55.18 ± 10.62	57.45 ± 11.26	1.02 (0.99–1.05)	0.183	—	55.90 ± 10.56	56.72 ± 7.19	1.01 (0.97–1.05)	0.701
Body mass index (kg/m^2^)^e^	<18.5	205	197	8	0.80 (0.25–1.35)	0.435	93	92	1	2.28 (1.57–2.98)	0.022
	18.5–25.0	1281	1255	26			539	523	16		
	>25.0	242	236	6			109	101	8		
Smoking (pack-y)			13.99 ± 20.81	22.25 ± 24.04	1.01 (1.00–1.03)	0.015	—	14.23 ± 20.53	13.64 ± 25.80	1.00 (0.98–1.02)	0.889
ASA score	1 + 2	1539	1508	31	Ref	0.022	664	643	21	Ref	0.355
	3 + 4	189	180	9	2.43 (1.67–3.19)		77	73	4	1.68 (0.58–2.77)	
COPD	Yes	61	56	5	4.16 (3.19–5.14)	0.004	20	16	4	8.33 (7.16–9.51)	0.000
	No	1667	1632	35	Ref		721	700	21	Ref	
Comorbidities excepting COPD	Yes	480	467	13	1.26 (0.59–1.93)	0.501	203	192	11	2.14 (1.34–2.95)	0.064
	No	1248	1221	27	Ref		538	524	14	Ref	
Anemia^a^	No	1086	1065	21	Ref	0.174	473	461	12	Ref	0.134
	Yes	642	623	19	1.55 (0.92–2.18)		278	265	13	1.84 (1.04–2.64)	
Albumin (g/L)	≥35	1361	1336	25	Ref	0.013	573	554	19	Ref	0.872
	<35	367	352	15	2.28 (1.63–2.93)		168	162	6	1.08 (0.15–2.01)	
Pre-operative blood transfusion	Yes	161	151	10	3.39 (2.66–4.13)	0.001	62	60	2	0.95 (−0.52–2.42)	0.946
	No	1567	1537	30	Ref		679	656	23	Ref	
Operation procedure	Open	1339	1301	38	5.65 (4.23–7.08)	0.017	565	544	21	1.66 (0.58–2.74)	0.359
	Laparoscopy	389	387	2	Ref		176	172	4	Ref	
Extent of gastrectomy	Total	415	400	15	1.93 (1.28–2.58)	0.047	166	158	8	1.66 (0.80–2.52)	1.359
	Subtotal^b^	1313	1288	25	Ref		575	558	17	Ref	
Combined multi-organ resection	Yes	162	151	11	3.86 (3.15–4.57)	0.000	58	54	4	2.34 (1.23–3.44)	0.132
	No	1566	1537	29	Ref		683	662	21	Ref	
Operation time (hour)	>4	369	352	17	2.81 (2.17–3.44)	0.002	165	153	12	3.40 (2.59–4.20)	0.003
	≤4	1359	1336	23	Ref		576	563	13	Ref	
Amount of bleeding (ml)	>300	341	328	13	2.00 (1.32–2.67)	0.044	153	146	7	1.52 (0.63–2.41)	0.359
	≤300	1387	1360	27	Ref		588	570	18	Ref	
Intra-operative blood transfusion	Yes	141	130	11	4.55 (3.83–5.26)	0.000	56	54	2	1.07 (−0.41–2.54)	0.932
	No	1587	1558	29	Ref		685	662	23	Ref	
Tumor location	Upper third	145	137	8	3.90 (3.04–4.76)	0.002	71	67	4	1.83 (0.70–2.95)	0.293
	Middle third	431	416	15	2.41 (1.70–3.11)	0.014	164	159	5	0.96 (−0.06–1.98)	0.942
	Lower third	1152	1135	17	Ref		506	490	16	Ref	
Tumor size (cm)^e^	<2.5	373	370	3	2.06 (1.56–2.55)	0.004	144	139	5	1.13 (0.51–1.75)	0.692
	2.5–5.0	949	928	21			431	418	13		
	>5.0	406	390	16			166	159	7		
TNM stage^c,e^	I	449	444	5	1.38 (0.97–1.79)	0.124	200	195	5	1.41 (0.89–1.92)	0.194
	II	365	354	11			149	146	3		
	III	914	890	24			392	375	17		

ASA: American Society of Anesthesiologist; COPD: chronic obstructive pulmonary disease. Ref: reference.

^a^Defines as hemoglobin level <120 g/L in male and <110 g/L in female.

^b^Subtotal contains two kinds of extent of gastrectomy: distal and proximal subtotal.

^c^Tumor stages are based on 7th edition of the Union for International Cancer Control TNM classification.

^d^OR: Odds Ratio, it was derived from univariate logistic analysis. CI: confidence interval

^e^These factors are continuous variables, and in univariate logistic analysis there are no reference category groups.

A total of 293 complications occurred in 220 patients of the entire cohort (8.91%), with pneumonia ranked as the second most common (n = 65, 2.63%), following surgical site infections (n = 116, 4.70%). With respect to the 65 patients who suffered from POI, the majority was managed with antibiotics and other conservative treatments (n = 50, 76.92%). Fifteen patients (23.08%) developed respiratory failure and needed intensive care unit care, which was significant more common than whose without POI (1.83%, *P* < 0.001). Moreover, 4 patients dead from respiratory failure and/or sepsis. The mortality rate was also significant higher in patients with POI than whose without POI (6.15% vs. 0.17%, *P* < 0.001). Additionally, patients with POI required longer post-operative hospital stays than those without POI (17.12 vs. 11.28 days, *P* < 0.001).

### Prognostic nomogram for POI

**Figure 2:**
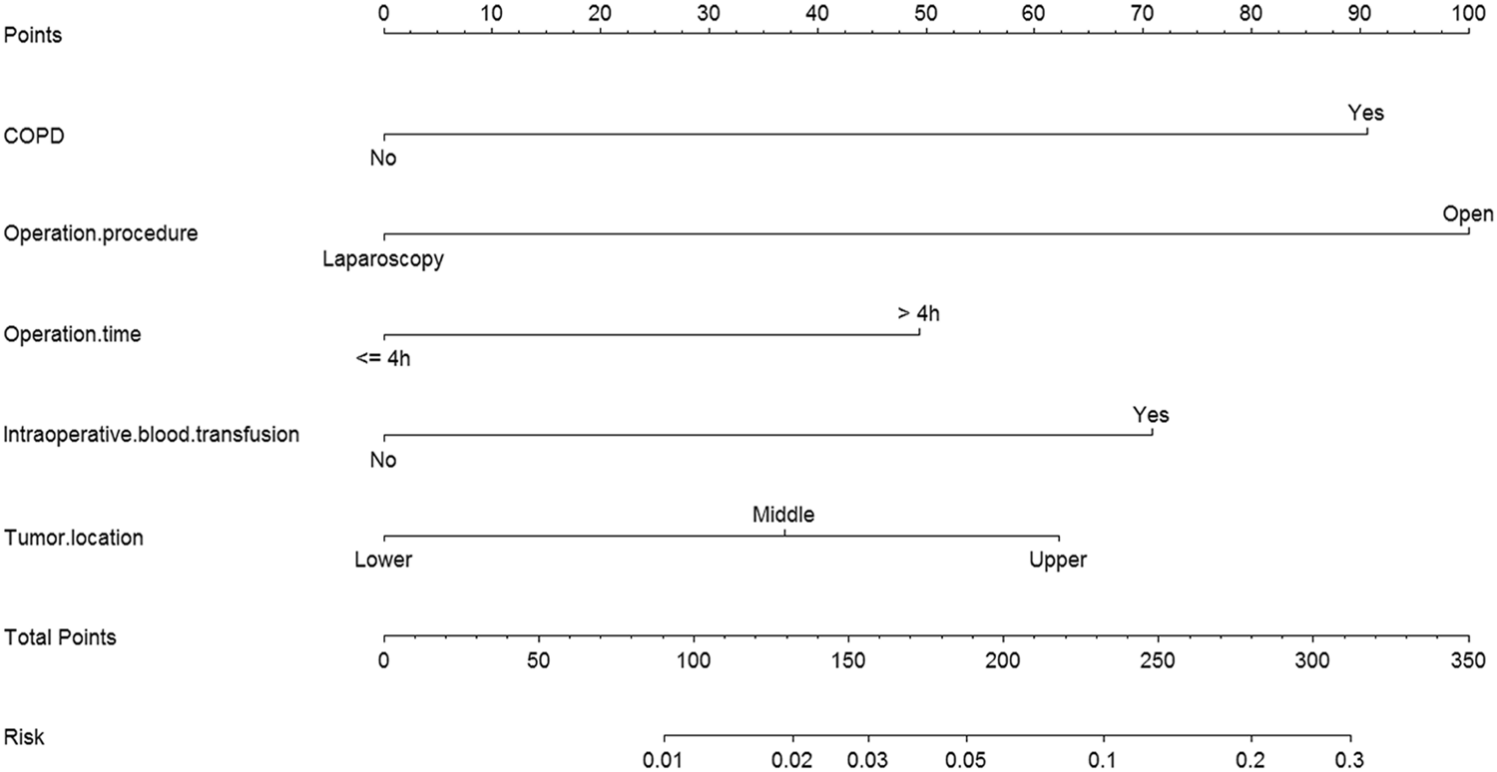
Prognostic nomogram for postoperative pulmonary infection in gastric cancer patients COPD, chronic obstructive pulmonary disease.

The univariate logistic analyses showed that smoking, American Society of Anesthesiologist (ASA) score, chronic obstructive pulmonary disease (COPD), serum albumin level, pre-operative blood transfusion, operation procedure, extent of gastrectomy, combined multi-organ resection, operation time, amount of bleeding, intra-operative blood transfusion, tumor location, and tumor size were associated with POI (*P* < 0.05 for all, Table 1). Further multivariate logistic regression analyses showed that COPD, open operative procedure, operation time ≥4 h, intra-operative blood transfusion, and tumor located at middle third were independent risk factors for POI (Table 2). The prognostic nomogram for POI was constructed based on these five prognostic factors (Figure 2). The nomogram plot of GC patient outcomes showed that compared with laparoscopic procedure, open operation was the greatest contributing factor to the risk of POI. Other factors that significantly contributed to POI risk to a lesser degree were COPD, intra-operative blood transfusion, tumor located at upper and/or middle third and operation time ≥4 h in descending order.

**Table 2:** Multivariate logstic analyses of risk factors for postoperative pulmonary infection in the development set.

Variable		Odds Ratio	95% CI	*P*	Score^a^
Chronic obstructive pulmonary disease	No	Ref	Ref		0
	Yes	4.63	3.62–5.65	0.003	91.0
Operation procedure	Laparoscopy	Ref	Ref		0
	Open	5.42	3.98–6.86	0.021	100.0
Operation time (h)	≤4	Ref	Ref		0
	>4	2.31	1.63–2.98	0.016	49.0
Intra-operative blood transfusion	No	Ref	Ref		0
	Yes	3.31	2.56–4.06	0.002	71.0
Tumor location	Lower third	Ref	Ref		0
	Middle third	1.86	1.14–2.60	0.025	37.0
	Upper third	2.86	1.98–3.75	0.092	62.5

CI: confidence interval; Ref: reference.

^a^Score was derived from the nomogram plot (Figure 2).

### Calibration and validation of the nomogram

**Figure 3:**
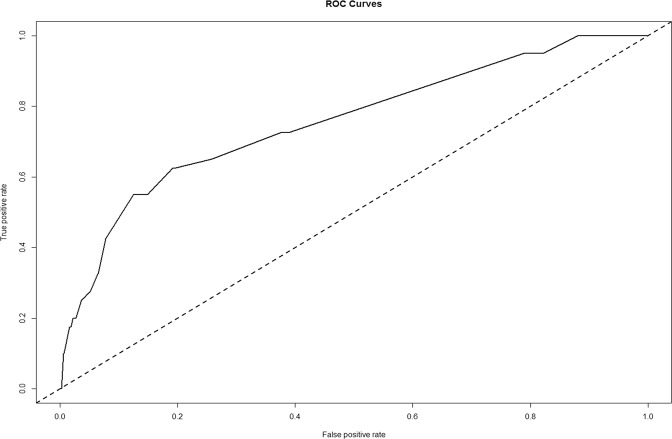
ROC curve of the predictive model for the training dataset. (ROC curve with an AUC value of 0.756). ROC, receiver-operating characteristic curve; AUC, area under the ROC curve.

**Figure 4:**
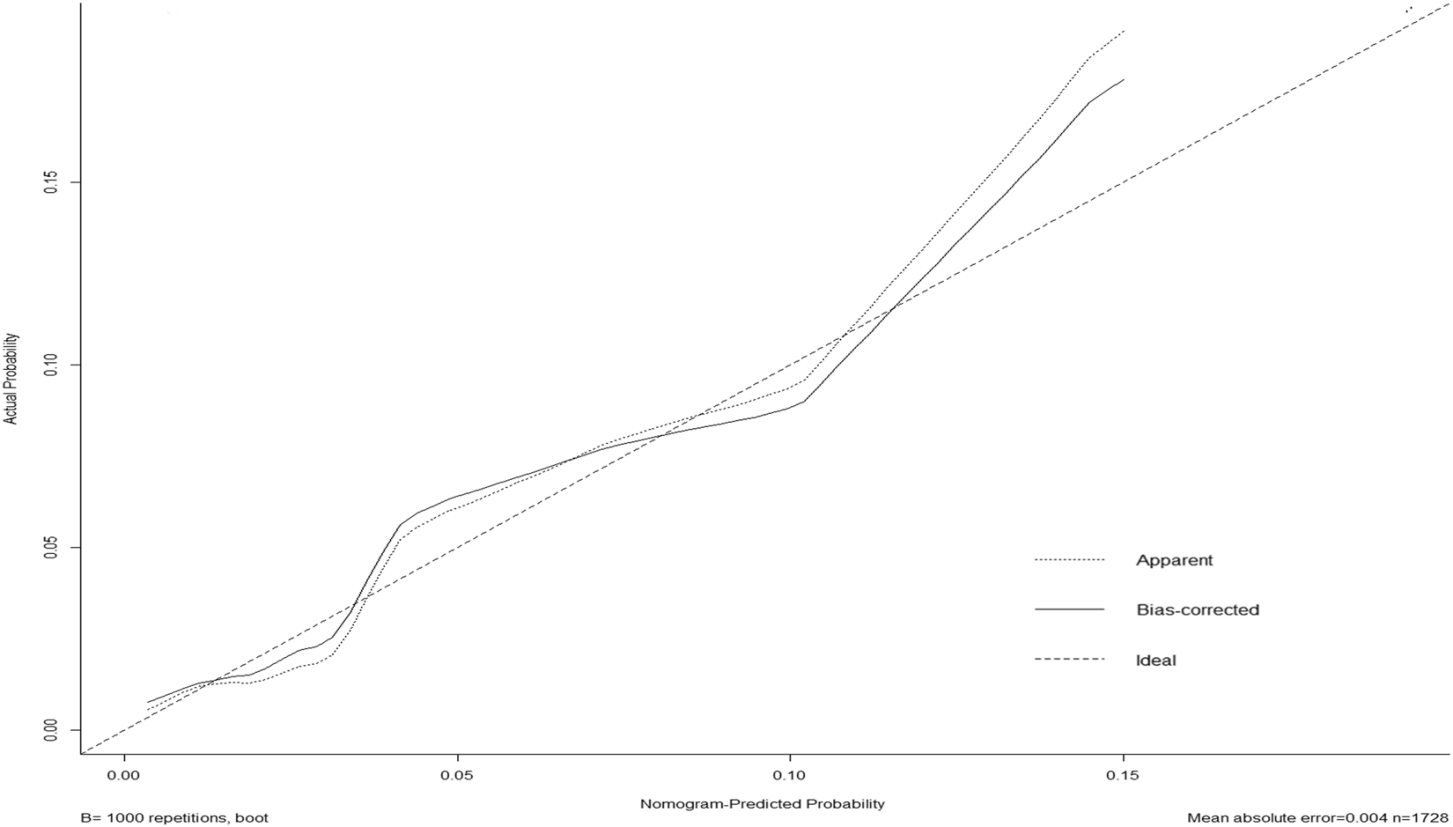
The calibration curves for predicting postoperative pulmonary infection in gastric cancer patients.

Internal validation of the nomogram model yielded a C-index value of 0.756 (95% CI: 0.675−0.837), which was identical to the AUC of the ROC plot (Figure 3). The internal calibration curve showed good agreement between nomogram predictions and actual observations with the predictive probability ranging from 0 to 0.15 (Figure 4). Data for the validation group (n = 741) were used for cross validation of the nomogram model, which yielded a C-index value of 0.652 (95% CI: 0.602−0.703), which was lower than the index of the development group.

## Discussion

In this retrospective study including a large cohort of GC patients who underwent curative gastrectomy from a single high-volume tertiary center in China, we found that the incidence of POI following radical gastrectomy for GC was 2.63%, which was similar to previous findings ranging from 1.1% to 3.6%^3,4,7^. Whereas some other studies reported that old patients (≥75 years) had a significant higher incidence of POI (5.1–13.3%), compared with that of patients aged 45 to 65 years^16,21,22^. Patients who suffered from POI had a significant higher rate of requiring intensive care and mortality rate, and longer post-operative hospital stays than those without POI. Given the significantly adverse influence of POI on post-operative recovery, surgeons should prioritize peri-operative managements to decrease its incidence. Although several studies have investigated the incidence and risk factors for POI following gastrectomy for GC, but the findings of these studies were usually based on a limited number of patients and limited variables, which may affect the adequate accuracy and power of the model^7,23^. Moreover, their conclusions have never been verified by other studies. Our logistic regression analysis found that open procedure, COPD, intra-operative blood transfusion, tumor located at  middle third and longer operation time were significant prognosticators of POI. Thus these five parameters were incorporated into the nomogram for predicting POI. Further internal and cross validation showed good agreement between nomogram predictions and actual observations with the predictive probability. To our knowledge, this is the first time to develop and crossly validate a nomogram for predicting POI in GC patients who underwent radical gastrectomy. This nomogram may serve as intuitive and simple tool for clinicians to identify those who are at greatest risk of suffering from POI, and take some active medical interventions for its prevention.

Our nomogram showed that open operation procedure accounted for the largest contribution to the risk of POI. Compared with open surgery, minimally invasive surgery has been shown to decrease blood loss, reduce time to ambulation, and quicker recovery. A smaller incision in laparoscopic surgery could relieve pain from coughing after surgery. Severe pain often influences patients’ compliance with incentive spirometry and thus decreases effective discharge of sputum and increases the risk of POI. Severe pain also could increase requirement for narcotics, which further suppresses patients’ respiratory drives and increases atelectasis^24^. A resent meta-analysis has confirmed that laparoscopic gastrectomy was associated with a lower incidence of pneumonia in patients with one or more high-risk factors, such as age ≥70 years, BMI ≥ 30 kg/m^2^, compared with open surgery^25^. In this study, the proportion of laparoscopic or laparoscopy-assisted gastrectomy increased from 4.04% (43/1065) to 37.18% (522/1404) through the first 4 years (from November 2010 to December 2014) to the last 4 years (from January 2015 to July 2018, *P* < 0.001), with the incidence of POI decreased from 3.66% to 1.85% (*P* = 0.005). Laparoscopic surgery has been widely performed in general clinical practice to manage early stage GC. And to date, it has been proved to be safe and have comparable oncological results in highly selective patients with advanced GC performed by experienced surgeons^18,26^. Thus, laparoscopic surgery may serve as an option to reduce POI in selective GC patients, although further prospective studies are needed.

Then, the contributions of the COPD, intra-operative blood transfusion, tumor location, and operation time in the nomogram, though significant, were less than that of operation procedure. Patients with COPD often have reduced respiratory function. Smoking is the most common cause of COPD, with factors such as air pollution and genetics playing a smaller role. Long exposure of these toxic particles or gases can induce the release of inflammatory mediators, inhibit ciliary movement in the airway, reduce the activity of pulmonary surface-active substances, increase airway mucus secretion, and reduce the ability of bronchial mucosa clearance, which ultimately increase the incidence of post-operative pulmonary complications^27^. Li *et al*. reported that COPD were an independent risk factor for post-operative complications in elderly GC patients group (OR = 3.756)^28^. For those with high-risk of developing POI, such as COPD, pre-operative smoking cessation, lung expansion modalities including deep breathing exercises and cough, and selective nasogastric decompression might reduce its incidence and thus, should be performed as possible^29^.

Intra-operative blood transfusion has been shown to increase susceptibility to pulmonary infection^7,16,21,28^. Transfusion induced systemic inflammation and transfusion-related immunomodulation (TRIM) was accepted as the underlying mechanism^19^. With respect to transfusion time, our previous study has also found that intra-operative, rather than pre-operative or post-operative blood transfusion, was independent risk for post-operative infections following gastrectomy^19^. Possible explanation was intra-operative blood transfusion may act synergistically with surgical stress to induce immuno-suppression. Therefore, to reduce POI, there could be some room for consideration to perform blood transfusion pre-operatively or post-operatively rather than intra-operatively. Given the relatively low incidence of POI and only 65 patients who developed POI in the present study, we did not investigate the association of the transfusion timing with POI. Thus, the conclusions need further more investigations.

Patients with tumors located in the upper third of the stomach were most vulnerable to POI, followed in descending order by the middle and lower third. The diaphragm, the most important breathing muscle, lies above the stomach. Based on the anatomical position, the closer the tumor was to the diaphragm, the greater extent to which the diaphragm reflex was suppress during gastric resection, which lowered lung activity by limiting spontaneous sighs and deep breaths, reducing effective coughing to discharge the sputum, and increasing lung secretion retention, which could increase the risk of POI. In addition, upper third GC was usually diagnosed later and accompanied by greater lymph node metastasis and deeper infiltration, and No. 110 and 111 lymph node (lower thoracic para-esophageal node) was recommended to dissection in some patients with tumors invading the esophagus^18^. Total gastrectomy was commonly performed in these patients, which was a more complex procedure than distal subtotal gastrectomy, thus requiring longer operation time and was conformed to be associated with higher incidence of morbidity, which may also increase the risk of POI^18,30^.

Prolonged surgery was identified as an independent risk factor for POI in our study, which was echoed by a study including 25419 colectomies, in which a regression model demonstrated that each 60-min increase in operative time was associated with at least 13% increased odds of post-operative pulmonary complications^24^. Interestingly, despite a potential for prolonged surgery, a laparoscopic procedure decreased half the absolute risk of post-operative pulmonary complications. Thus, the authors concluded that when safe, laparoscopy should be performed as possible. Additionally, prolonged surgery probably means technical difficulty, such as overweight patients, extended lymphadenectomy or combined resection, which may also increase the risk of morbidity. Using ultrasonically activated coagulating shears, improving surgical experience and work volume may reduce the operative time, and thus probably decrease the risk of POI^31,32^.

Nomogram validation is highly essential for confirming the predictive performance and determining the generalizability of the nomogram to other GC patients^33^. We developed an internal validation method for our nomogram model, and found that the C-index value was 0.756 (95% CI: 0.675−0.837), which was identical to the AUC of the ROC plot. The calibration curve showed good agreement between the nomogram predictions and actual observations within a predictive probability range of 0 to 0.15, which indicated satisfactory and reliable nomogram performance. To our knowledge, this is the first report that POI risk nomogram have been developed using these parameters. Our nomogram allows surgeons to easily and accurately identify GC patients who are at high risk of POI, then to optimize treatment for POI prevention. From these significant factors for POI in the model, doctors are encouraged to perform laparoscopic surgery for selective patients if possible, especially for those with high-risk factors, such as COPD and upper third GC. Using ultrasonically activated coagulating shears and improving surgical experience to reduce operation time, performing blood transfusion pre-operatively instead of intra-operatively, may also decrease the risk of POI following gastrectomy for GC, which needs to be verified by further studies.

There are also some limitations of our findings. These include the retrospective nature and single-institution design of our study. Thus selective bias was impossible to avoid and the conclusion should be cited with appropriate interpretation. Second, the predictive factors involved in our nomogram were acquired from routine demographic and laboratory testing. Other important parameters, such as respiratory function testing and data on immune function, were not considered in the design of our nomogram. Third, the exact onset time of POI was not investigated in the present study, whereas early or late occurrence, “primary” POI or POI following other complications (such as anastomotic leakage) has been identified to be associated with different risk factors^34^. Fourth, Western GC patients usually have higher BMI, longer operation time and with different process of healthcare during hospitalization (such as nasogastric decompression and prophylactic antibiotic usage) and post-discharge compared with those in China, which may affect the applicability of the nomogram in Western patients. Last, but not the least, the sample size for the dataset used for nomogram cross validation was relatively small, and future prospective studies utilizing larger sample sizes are warranted to confirm our findings.

## Conclusions

The GC nomogram for predicting the risk of POI has been established and validated. Our results confirmed that open operation, COPD, intra-operative blood transfusion, tumor located at upper and/or middle third and operation time ≥4 h were significant factors in the nomogram to predict POI. This makes it possible for physicians to choose some clinical activities to reduce post-operative pulmonary infection accurately and simply by using the picture tool of our nomogram.
